# Cytotoxicity and Modes of Action of the Methanol Extracts of Six Cameroonian Medicinal Plants against Multidrug-Resistant Tumor Cells

**DOI:** 10.1155/2013/285903

**Published:** 2013-09-23

**Authors:** Victor Kuete, Aimé G. Fankam, Benjamin Wiench, Thomas Efferth

**Affiliations:** ^1^Department of Pharmaceutical Biology, Institute of Pharmacy and Biochemistry, Johannes Gutenberg University of Mainz, Staudinger Weg 5, 55128 Mainz, Germany; ^2^Department of Biochemistry, Faculty of Science, University of Dschang, P.O. Box 67, Dschang, Cameroon

## Abstract

*Introduction*. The present study aims at evaluating the cytotoxicity of twelve parts from six Cameroonian medicinal plants on sensitive and drug-resistant cancer cell lines. We also studied the mode of action of the most active plants, *Gladiolus quartinianus*, *Vepris soyauxii*, and *Anonidium mannii*. *Methods*. The cytotoxicity of the extracts was determined using a resazurin assay. Flow cytometry was used for cell-cycle analysis and detection of apoptosis, analysis of mitochondrial membrane potential (MMP), and measurement of reactive oxygen species (ROS). *Results*. At 40 g/mL, three extracts showed a growth of CCRF-CEM leukemia cells by less than 50%. This includes the extracts from *G. quartinianus* (GQW; 25.69%), *Vepris soyauxii* leaves (VSL; 29.82%), and *Anonidium mannii* leaves (AML; 31.58%). The lowest IC_50_ values below 30 **μ**g/mL were obtained with GQW, AML and VSL against 7/9, 8/9, and 9/9 tested cancer cell lines, respectively. The lowest IC_50_ values for each plant were 4.09 **μ**g/mL, and 9.14 **μ**g/mL (against U87MG.Δ*EGFR* cells), respectively, for VSL and AML and 10.57 **μ**g/mL (against CCRF-CEM cells) for GQW. GQW induced cell cycle arrest between G0/G1 and S phases, whilst VSL and AML induced arrest in G0/G1. All three extracts induced apoptosis in CCRF-CEM cells by loss of MMP, whilst AML also enhanced production of ROS. *Conclusion*. The three active plants may be a source for the development of new anticancer drugs.

## 1. Introduction

Cancer is one of the major causes of death in humans representing the third leading cause of death worldwide (12.4%), the first being cardiovascular disease (30%) and the second being infectious diseases, including HIV/AIDS (18.8%) [1]. Chemotherapy remains the treatment of choice in many malignant diseases [2]. Nevertheless, the appearance of drug resistance, in particular multidrug resistance (MDR), can make many of the clinically established anticancer drugs ineffective [3]. Thus, MDR is one of the major concerns preventing cure of many cancer patients. Also, malignancies are increasingly recognized as a critical public health problem in Africa [4]. Worldwide, the number of new cancer cases will annually reach 15 million by 2020, 70% of which will occur in developing countries, where governments are less prepared to address the growing cancer burden and where survival rates are often less than half of those in more developed countries [4]. It has been observed that throughout the continent, though infectious diseases continue to burden African population, noninfectious diseases require much more attention [4]. Currently, limited funding is available to tackle cancer in African countries. Awareness of this impeding epidemic in Africa deserves priority, and further resources should be mobilized to both prevent and treat cancer. Research on anticancer agents has become a worldwide effort in both developed and developing countries, since chemotherapy is a mainstay in the treatment of many malignancies [5]. The majority of standard anticancer drugs has been isolated or derived from natural sources, based on their use in traditional medicine [6]. Screenings of medicinal plants used as anticancer drugs have provided modern medicine with effective cytotoxic pharmaceuticals. More than 60% of the approved anticancer drugs in USA were from natural origin [7–9]. In Cameroon, the use of plants in traditional medicine systems has been extensively documented in the Cameroonian pharmacopoeia [10]. Evidence highlighting the importance of these plants for cancer therapy has been provided [11–16]. However, whether these plants are also effective in cells resistant to standard chemotherapy is largely unknown. It has been recommended that ethnopharmacological usages such as immune and skin disorders, inflammatory, infectious, parasitic, and viral diseases should be taken into account when selecting plants used to treat cancer, since these reflect disease states bearing relevance to cancer or cancer-like symptoms [17, 18]. Though the plants selected in the present studied are used in the Cameroonian traditional medicine to fight cancers, there is still a lack of published data regarding the use. Therefore, in our continuous search of the cytotoxic candidates from Cameroonian plants with unpublished ethnopharmacological information related to cancer use, we investigated the antiproliferative potential of six Cameroonian plants against cancer cell lines with different mechanisms of drug resistance, that is, ATP-binding cassette (ABC) transporters (P-glycoprotein, breast cancer resistance protein), tumor suppressors (p53), or oncogenes (epidermal growth factor receptor). The most cytotoxic extracts from *Gladiolus quartinianus* A. Rich. (Iridaceae), *Vepris soyauxii* Engl. (Rutaceae) and *Anonidium mannii* (oliv) Engl. et Diels. (Annonaceae) were further analyzed to study their mode of action regarding cell-cycle distribution, MMP, and ROS.

## 2. Materials and Methods

### 2.1. Plant Material

All medicinal plants used in the present work were collected in different areas of Cameroon between January and April 2012 (Table 1). The plants were identified at the National Herbarium (Yaounde, Cameroon), where voucher, specimens were deposited under the references numbers given in Table 1.

### 2.2. Extraction

The air-dried and powdered plant samples (1 kg) were soaked in methanol (3 L) for 48 h, at room temperature. The methanol extracts were concentrated *in vacuum* to obtain the crude extracts [12]. These extracts were then stored at 4°C until further use.

### 2.3. Chemicals

Doxorubicin, vinblastine, and daunorubicin were provided by the University Pharmacy of the Johannes Gutenberg University (Mainz, Germany) and dissolved in PBS (Invitrogen, Eggenstein, Germany) at a concentration of 10 mM. Geneticin (72.18 mM) was purchased from Sigma-Aldrich (Munich, Germany).

### 2.4. Preliminary Phytochemical Investigations

The major secondary metabolites classes such as alkaloids, anthocyanins, anthraquinones, flavonoids, phenols, saponins, sterols, and triterpenes (Table 2) were determined according to a common phytochemical methods previously described [34].

### 2.5. Cell Cultures

Drug-sensitive CCRF-CEM and multidrug-resistant CEM/ADR5000 leukemia cells were maintained in RPMI 1640 medium (Invitrogen) supplemented with 10% fetal calf serum in a humidified 5% CO_2_ atmosphere at 37°C. Sensitive and resistant cells were kindly provided by Dr. Axel Sauerbrey (Department of Pediatrics, University of Jena, Jena, Germany). The generation of the resistant subline was described [35]. The specific overexpression of P-glycoprotein, but not other ABC transporters, has been reported [36, 37]. Breast cancer cells transduced with control vector (MDA-MB-231-pcDNA3) or with cDNA for the breast cancer resistance protein, *BCRP *(MDA-MB-231-*BCRP *clone 23) were maintained under standard conditions as described previously for CCRF-CEM cells. Human wild-type HCT116 (*p53* ^+/+^) colon cancer cells as well as knockout clones HCT116 (*p53* ^−/−^) derived by homologous recombination were a generous gift from Dr. B. Vogelstein and H. Hermeking (Howard Hughes Medical Institute, Baltimore, MD, USA). Human glioblastoma multiforme U87MG cells (nontransduced) and U87MG cell line transduced with an expression vector harboring an epidermal growth factor receptor (*EGFR*) gene with a genomic deletion of exons 2 through 7 (U87MG.Δ*EGFR*) were kindly provided by Dr. W. K. Cavenee (Ludwig Institute for Cancer Research, San Diego, CA, USA) [38]. MDA-MB-231-*BCRP, *U87MG.Δ*EGFR,* and HCT116 (*p53* ^−/−^) were maintained in DMEM medium containing 10% FBS (Invitrogen) and 1% penicillin (100 U/mL) streptomycin (100 *μ*g/mL) (Invitrogen) and were continuously treated with 800 ng/mL and 400 *μ*g/mL geneticin, respectively. Human HepG2 hepatocellular carcinoma cells and normal AML12 hepatocytes were obtained from American Type Culture Collection (ATCC, USA). The previous medium without geneticin was used to maintain MDA-MB-231, U87MG, HCT116 (*p53* ^+/+^), HepG2, and AML12 cell lines. The cells were passaged twice weekly. All experiments were performed with cells in the logarithmic growth phase. 

### 2.6. Resazurin Reduction Assay

Resazurin reduction assay [16, 39] was performed to assess cytotoxicity of the studied samples toward cancer cells. The assay is based on reduction of the indicator dye, resazurin, to the highly fluorescent resorufin by viable cells. Nonviable cells rapidly lose the metabolic capacity to reduce resazurin and thus produce no fluorescent signal. Briefly, adherent cells were detached by treatment with 0.25% trypsin/EDTA (Invitrogen, Darmstadt, Germany) and an aliquot of 1 × 10^4^ cells was placed in each well of a 96-well cell culture plate (Thermo Scientific, Langenselbold, Germany) in a total volume of 200 *μ*L. Cells were allowed to attach overnight and then were treated with different concentrations of the studied sample. For suspension cells, aliquots of 2 × 10^4^ cells per well were seeded in 96-wellplates in a total volume of 100 *μ*L. The studied sample was immediately added in varying concentrations in additional 100 *μ*L of culture medium to obtain a total volume of 200 *μ*L/well. After 24 h or 48 h, 20 *μ*L resazurin (Sigma-Aldrich, Schnelldorf, Germany) 0.01% w/v in ddH_2_O was added to each well and the plates were incubated at 37°C for 4 h. Fluorescence was measured on an Infinite M2000 Pro plate reader (Tecan, Crailsheim, Germany) using an excitation wavelength of 544 nm and an emission wavelength of 590 nm. Each assay was done at least two times, with six replicate each. The viability was evaluated based on a comparison with untreated cells. IC_50_ values represent the sample's concentrations required to inhibit 50% of cell proliferation and were calculated from a calibration curve by linear regression using Microsoft Excel.

### 2.7. Flow Cytometry for Cell Cycle Analysis and Detection of Apoptotic Cells

Cell-cycle analysis was performed by flow cytometry using The Vybrant DyeCycle (Initrogen). The Vybrant DyeCycle Violet stain is a DNA-selective, cell membrane-permeant, and nonfluorescent dye for DNA content analysis in living cells. The Vybrant DyeCycle Violet stain is fluorescent upon binding to double-stranded DNA. Leukemia CCRF-CEM cells (1 × 10^6^) were treated with the concentrations equivalent to the IC_50_ values of the crude extract for 24 h, 48, and 72 h. Following incubation, 1 *μ*L of Vybrant DyeCycle Violet stain was added to 1 mL of cell suspension and incubated for 30 min at 37°C. Cells were measured on an LSR-Fortessa FACS analyzer (Becton-Dickinson, Germany) using the violet laser. Ten thousand cells were counted for each sample. Vybrant DyeCycle Violet stain was measured with 440 nm excitation. Cytographs were analyzed using FlowJo software (Celeza, Switzerland). All experiments were performed at least in triplicate.

### 2.8. Analysis of Mitochondrial Membrane Potential (MMP)

The effects of extract on the MMP were analyzed by 5,5′,6,6′-tetrachloro-1,1′,3,3′-tetraethylbenzimidazolylcarbocyanine iodide) (JC-1; Biomol, Germany) staining. JC-1 is a dye that can selectively enter into mitochondria and exhibits an intense red fluorescence in healthy mitochondria with normal membrane potentials. In cells with reduced MMP, the red fluorescence disappears. Briefly, 1 × 10^6^ CCRF-CEM cells treated with different concentrations of the test compounds or DMSO (solvent control) for 24 h were incubated with JC-1 staining solution according to the manufacturer's protocol for 30 min. Subsequently, cells were measured in an LSR-Fortessa FACS analyzer (Becton-Dickinson). For each sample, 1 × 10^4^ cells were counted. The JC-1 signal was measured with 561 nm excitation (150 mW) and detected using a 586/15 nm bandpass filter. The compounds signal was analyzed with 640 nm excitation (40 mW) and detected using a 730/45 nm bandpass filter. All parameters were plotted on a logarithmic scale. Cytographs were analyzed using FlowJo software (Celeza, Switzerland). All experiments were performed at least in triplicate.

### 2.9. Measurement of Reactive Oxygen Species (ROS) by Flow Cytometry

2′,7′-Dichlorodihydrofluorescein diacetate (H_2_DCFH-DA) (Sigma-Aldrich, Germany) is a probe used for the highly sensitive and quantifiable detection of ROS. The nonfluorescent H_2_DCFH-DA diffuses into the cells and is cleaved by cytoplasmic esterases into 2′,7′-dichlorodihydrofluorescein (H_2_DCF) which is unable to diffuse back out of the cells. In the presence of hydrogen peroxide, H_2_DCF is oxidized to the fluorescent molecule dichlorofluorescein (DCF) by peroxidases. The fluorescent signal emanating from DCF can be measured and quantified by flow cytometry, thus providing an indication of intracellular ROS concentration [40, 41]. Briefly, 2 × 10^6^ CCRF-CEM cells were resuspended in PBS and incubated with 2 *μ*M H_2_DCFH-DA for 20 min in the dark. Subsequently, cells were washed with PBS and resuspended in RPMI 1640 culture medium containing different concentrations of extract or DMSO (solvent control). After 1 h of incubation, cells were washed and suspended in PBS. Subsequently, cells were measured in an FACS Calibur flow cytometer (Becton-Dickinson, Germany). For each sample 1 × 10^4^ cells were counted. DCF was measured at 488 nm excitation (25 mW) and detected using a 530/30 nm bandpass filter. All parameters were plotted on a logarithmic scale. Cytographs were analyzed using FlowJo software (Celeza, Switzerland). All experiments were performed at least in triplicate.

## 3. Results 

### 3.1. Chemical Composition of the Studied Extracts

The results of the qualitative analysis showed that each of the studied plant extract contained at least one class of secondary metabolites such as alkaloids, anthocyanins, anthraquinones, flavonoids, phenols, saponins, and triterpenes. All studied extracts contained alkaloids, phenols, and tannins (Table 2). 

### 3.2. Cytotoxicity of the Studied Samples

**Figure 1:**
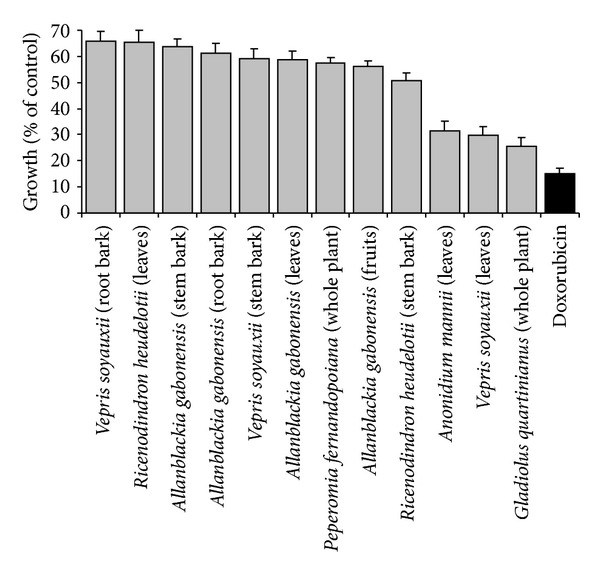
Growth (% of untreated control) of CCRF-CEM leukemia cells in the presence of plant extracts (40 *μ*g/mL) or doxorubicin (10 *μ*g/mL).

The growth inhibition of CCRF-CEM cells induced by 12 extracts belonging to six medicinal plants is depicted in Figure 1. The extracts from *Gladiolus quartinianus* (whole plant; GQW; 25.69%), *Vepris soyauxii* (leaves; VSL; 29.82%), and *Anonidium mannii* (leaves; AML; 31.58%) inhibited cell growth by more than 50% at 40 *μ*g/mL. 

To investigate these extracts in more detail, their IC_50_ values were determined in a panel of cancer cell lines. The VSL extract was active (IC_50_ < 40 *μ*g/mL) against all 9 sensitive or drug-resistant cell lines. IC_50_ values below 30 *μ*g/mL were obtained with GQW, AML, and VSLagainst 7/9, 8/9, and 9/9 tested cancer cell lines, respectively. The IC_50_ values were in a range from 4.09 *μ*g/mL (U87MG.Δ*EGFR* cells) to 13.60 *μ*g/mL (HepG2 cells) for VSL from 10.57 *μ*g/mL (CCRF-CEM) to 34.01 *μ*g/mL (U87MG.Δ*EGFR*) for GQW, and from 9.14 *μ*g/mL (U87MG.Δ*EGFR*) to 32.02 *μ*g/mL (MDA-MB-231-*BCRP*) for AML. For the control drug doxorubicin, the IC_50_ values were in a range from 0.11 *μ*g/mL (CCRF-CEM cells) to 195.12 *μ*g/mL (CEM/ADR5000 cells) (Table 3). High degrees of resistance to doxorubicin were observed for CEM/ADR 5000 cells (1772-fold), MDA-MB-231-*BCRP* cells (7.11-fold), and U87MG.Δ*EGFR* (5.76-fold) compared to their corresponding parental cell lines. HCT116 (*p53* ^−/−^) cells were weakly resistant to doxorubicin (2.84-fold) compared to HCT116 (*p53* ^+/+^) cells. Interestingly, the drug-resistant cell lines were not or only weakly resistant to the tested extracts (≤2.53-fold). Remarkably, none of the tested extract inhibited the growth of more than 50% normal AML12 hepatocytes at a concentration of 40 *μ*g/mL. Collateral sensitivity, which means that resistant cells are more sensitive than sensitive cells, was observed with the three extracts against U87MG.Δ*EGFR* with degree of resistances below 1. This was also noted for the VSL and AML extracts against HepG2 cells and AML extract against CEM/ADR5000 cells. All the plant extracts showed higher IC_50_ values in normal AML12 hepatocytes compared to HepG2 liver cancer cells. Furthermore, AML12 normal hepatocytes were more doxorubicin resistant than HepG2 cancer cells towards doxorubicin. None of the extracts inhibited normal AML12 hepatocytes by more than 50%.

### 3.3. Cell Cycle Distribution and Apoptosis

**Figure 2:**
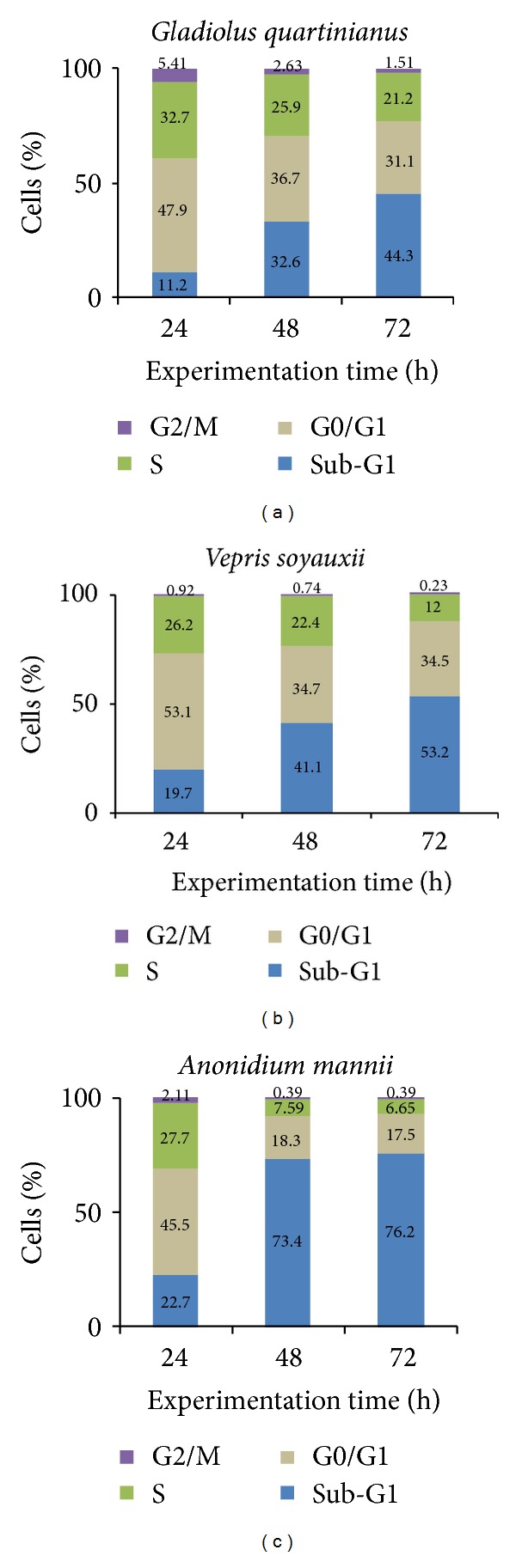
Cell-cycle distribution of CCRF-CEM cells treated with plant extractsordoxorubicin at their corresponding IC_50_ values for 72 h. Data of control and doxorubicin obtained under similar experimental conditions were previously reported [33]. Flow cytometry histograms are available as supportive information (Figure S1).

The cell-cycle distribution and induction of apoptosis of CCRF-CEM cells upon treatment with GQW, VSL AML, are depicted in Figure 2. Upon 72 h treatment, the GQWextract induced cell cycle arrest between G0/G1 and S phases whilst VSL and AMLextracts induced G0/G1 arrest. The three extracts led to a time-dependent increase of sub-G0/G1 cells, indicating induction of apoptosis. CCRF-CEM cells treated with concentrations equivalent to the IC_50_ value of each studied extracts progressively underwent apoptosis, with percentages in sub-G0/G1 phase ranging from 11.2% (24 h) to 44.3% (72 h) for GQW, from 19.7% (24 h) to 53.2% (72 h) for VSL, and from 22.7% (24 h) to 76.2% (72 h) for AML. The values of the sub-G0/G1 phase recorded with AMLwere higher than those obtained with nontreated cells (range from 3.82% (24 h) to 9.37% (72 h)), but were comparable to those obtained for the control drug, doxorubicin (range from 59.4% (24 h) to 71.9% (72 h)) (see Supplementary Material available online at http://dx.doi.org/10.1155/2013/285903, Figure  S1).

### 3.4. Effect on the Mitochondrial Membrane Potential (MMP)

**Figure 3:**
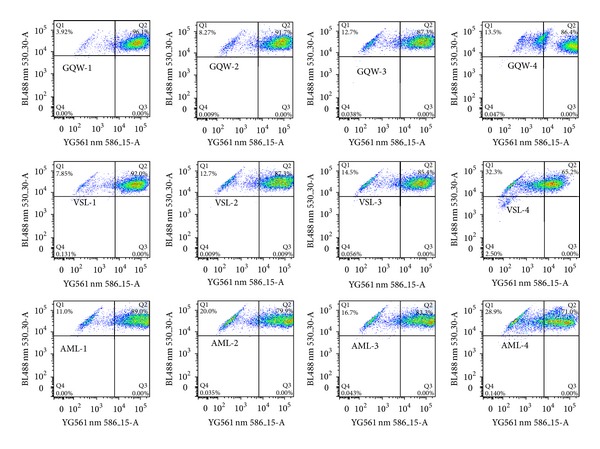
Effect of plant extracts and vinblastine (VIN) on the MMP of CCRF-CEM cells after 24 h of treatment. Data of control and vinblastine under similar experimental conditions were previously reported [33]. Samples were tested at their 1/4 × IC_50_ (1), 1/2 × IC_50_ (2), IC_50_ (3), and 2 × IC_50_ (4) values. The IC_50_ values are 0.20 *μ*M for VIN, 10.57 *μ*g/mL (*Gladiolus quartinianus *whole plant,* GQW*), 9.28 *μ*g/mL (*Vepris soyauxii *leaves,* VSL*), and 17.32 *μ*g/mL (*Anonidium mannii *leaves,* AML*).

We assessed the effect of the GQW, VSL, and AML extracts on MMP in CCRF-CEM cells. As shown in Figure 3, percentage alterations of 13.5%, 28.9%, and 32.3% were induced by GQW, VSL, and AML extracts, respectively, after 24 h of treatment with twofold IC_50._ The MMP value for untreated cells was 4.81%. Under similar experimental conditions, these values were lower than that of the reference compound, vinblastine which yielded 48.6% as previously reported [33]. 

### 3.5. Effects on Reactive Oxygen Species (ROS)

**Figure 4:**
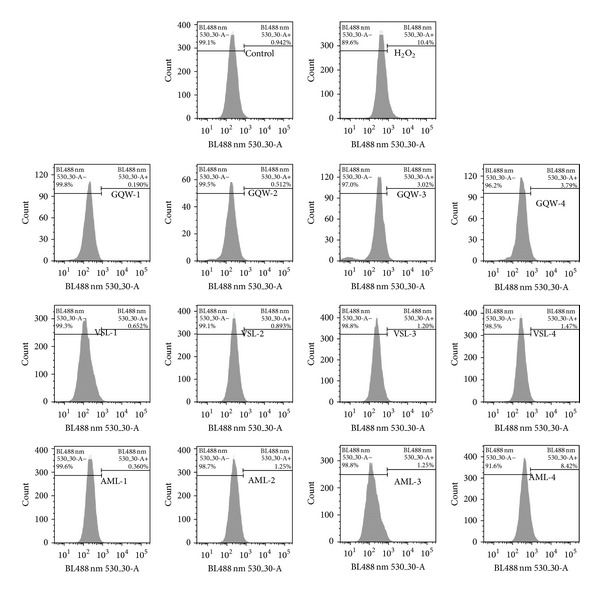
Effect of plant extracts and H_2_O_2_ (at 50 *μ*M) on the ROS production of CCRF-CEM cells after 24 h treatment. Samples were tested at their 1/4 × IC_50_ (1), 1/2 × IC_50_ (2), IC_50_ (3), and 2 × IC_50_ (4) values. The IC_50_ values are 10.57 *μ*g/mL (*Gladiolus quartinianus* whole plant,* GQW*), 9.28 *μ*g/mL (*Vepris soyauxii *leaves,* VSL*), and 17.32 *μ*g/mL (*Anonidium mannii *leaves,* AML*).

The effects of the GQW, VSL, and AMLextracts on ROS levels were investigated in CCRF-CEM cells after 24 h treatment (Figure 4). The control agent, H_2_O_2,_ increased ROS level to 10.4%, while ROS production in nontreated cells was 0.94%. Only AML induced significant ROS production in CCRF-CEM cells treated with a concentration equivalent to 2 × IC_50_ (8.42%). 

## 4. Discussion

Drug resistance is a complex multifactorial phenomenon that can result from a number of biochemical mechanisms, including decreased drug uptake or increased drug efflux, perturbed expression of target enzymes or altered target enzymes, altered metabolism of drugs, increased repair of drug-induced DNA damage, or failure to undergo apoptosis [42, 43]. Resistance phenomena may lead to failure of therapy with fatal outcome for cancer patients. Secondary metabolites play an important role in plant defense against herbivores, microbial infections, and other interspecies defenses and can be exploited to fight human diseases, including cancer [44]. Their antiproliferative properties have been broadly discussed [45]. In the present study, the classes of secondary metabolites detected in the tested plant extracts (Table 2) provide a preliminary explanation on their activities. The obtained results represent the first phytochemical data on the cytotoxic activity of *G. quartinianus*, *V. soyauxii*, and *A. manni*.

According to the criteria of the ATCC, 30 *μ*g/mL represent the upper IC_50_ limit considered promising for purification of a crude extract [46]. In the present work, the highest concentration tested (40 *μ*g/mL) in our screening was slightly above this limit. Herein, we recorded IC_50_ values below 30 *μ*g/mL for GQW, AML, and VSL extracts towards the majority of the tested cancer cell lines (Table 3). This demonstrates that the crude extracts of GQW, AML, and VSL could serve as potential sources of cytotoxic compounds. 

In addition to the identification of crude extracts with reasonable low IC_50_ values, we identified extracts capable of killing otherwise drug-resistant cancer cells. Having in mind that drug resistance is a major obstacle of chemotherapy in the clinic, the search for novel noncross-resistant cytotoxic compound from natural sources is urgently warranted. Drug-resistant cell models overexpressing P-glycoprotein, BCRP, or ΔEGFR as well as p53 knockout cells were used to assess the suitability of the studied extracts to tackle multifactorial drug resistance. The degrees of resistance of the three extracts were generally lower than that of doxorubicin in corresponding drug-resistant cell lines (Table 3), clearly highlighting their possible role fighting multidrug resistance. It was pleasing that even collateral sensitivity was observed in several cases. This phenomenon is characterized by the fact that drug-resistant cells are more sensitive to a test compound than the parental sensitive cells [47, 48]. 

The objective of cancer chemotherapy is to kill cancer cells with as little damage as possible to normal cells [49]. The GQW, VSL, and AML extracts were more cytotoxic towards HepG2 liver carcinoma cells and the other cancer cell lines tested than towards normal AML12 hepatocytes. This highlights at least some specificity of the three plant extracts towards target malignant cells with little effects on normal cells. 

 We further found that the GQW, VSL, and AML extracts induced apoptosis by disruption of MMP, whilst in addition AML produced ROS. To the best of our knowledge, the cytotoxicity of GQW, VSL, and AML is being reported here for the first time. Therefore, the isolation of the active constituents from these plants is worthwhile for the better understanding of their activities towards cancer cells. 

In conclusion, the present study provides evidence of the cytotoxic potential of GQW, VSL, and AML extracts on sensitive and drug-resistant cancer cell lines. The three extracts induced apoptosis in CCRF-CEM cells by loss of MMP and, in the case of AML, also enhanced ROS production. These plant extracts merit more detailed investigations to improve therapy of drug-resistant and refractory tumors in the future. 

## Supplementary Material

The three plants induced apoptosis and cell cycle arrest in leukemia CCRF-CEM cells. Upon 72 h treatment, *Gladiolus quartinianus* induced cell cycle arrest between G0/G1 and S phases, whilst *Vepris soyauxii* and *Anonidium mannii* induced arrest in G0/G1.

## Figures and Tables

**Table 1:** Pharmacognosy of Cameroonian medicinal plants.

Samples, family, and herbarium number^a^	Traditional treatment	Part used in this study and extraction yield (%)^b^	Area of plant collection	Known bioactive (or potentially active) compounds	Screened activity for crude plant extract
*Allanblackia gabonensis *Pellegr. (Clusiaceae); 17275 SRF/Cam	Dysentery, cold, toothache [19, 20]; pain, rheumatism, inflammations [21], cancer (personal information)	Fruits (4.14%), leaves (10.16%), stem (8.42%) and roots bark (8.69%)	Lebialem, South West region of Cameroon	Allan Xanthones A and D; 1,3,6,7-tetrahydroxy-2-(3-methylbut-2-enyl)xanthone [22]	Antimicrobial against Gram-positive and Gram-negative bacteria, yeasts, and mycelial fungus [22]; analgesic and anti-inflammatory effect of aqueous extract of the stem bark [21].
*Anonidium mannii* (oliv) Engl. et Diels. (Annonaceae); 1918/SRFK	Sore feet, spider bite, bronchitis, dysentery, sterility caused by poison, gastroenteritis [23]; syphilis, infectious diseases [24]; diarrhea, snake bite, malaria [25], cancer (personal information)	Leaves (3.39%)	Bafoussam, West region of Cameroon	Not reported	Not reported
*Gladiolus quartinianus *A. Rich (Iridaceae); 17260/SRF/Cam	Gastrointestinal infection, cancer (personal information)	Whole plant (10.22%)	Lebialem, South West region of Cameroon	Not reported	Not reported
*Peperomia fernandopoiana* C.DC. (Piperaceae); 7171 SRF/Cam	Gastrointestinal infection, cancer (personal information)	Whole plant (7.28%)	Lebialem, South West region of Cameroon	Not reported	Not reported
*Recinodindron heudelotii *(Baill.) ex Pax. (Euphorbiaceae); 19695 SRF/Cam	Cough, antidote, intestinal diseases, dysentery [26–28]; malaria, anaemia, stomach pain, easy delivery, yellow fever, aphrodisiac [29], cancer (personal information)	Leaves (5.18%) and stems bark (5.72%)	Melon, littoral region of Cameroon	*E*-ferulic acid octacosylate, 3-methylmethylorsellinate, lupeol, heudoletinone, 1,2-dihydroheudoleunol [26], aleuritolic acid 1 and labda-8(17),13-dien 3*β*,15-diol 2 [30]	Antimicrobial activity of the stem bark against *Streptococcus faecalis * [30], *Staphylococcus aureus, Bacillus cereus, Escherichia coli, Shigella dysenteriae, Shigella flexneri, Salmonella typhi, Pseudomonas aeruginosa, Klebsiella pneumoniae, Candida albicans* [31], antioxidant [32].
*Vepris soyauxii *Engl. (Rutaceae); 18394 SFR/Cam	Antifibromyoma, stomachache, malaria [32], cancer (personal information)	Leaves (10.16%), stems (5.18%), and roots bark (9.26%)	Melon, littoral region of Cameroon	Not reported	Not reported

^a^Plants were identified at the Cameroon National Herbarium (HNC); ^b^The percentage of the methanol extract.

**Table 2:** Chemical constituents and extraction yield of the studied plant extracts.

Studied samples	Phytochemical constituents								
	Alkaloids	Anthocyanins	Anthraquinones	Flavonoids	Phenols	Saponins	Tannins	Sterols	Triterpenes
*Allanblackia gabonensis *									
Leaves	+	+	−	+	+	−	+	−	−
Stem bark	+	+	+	+	+	+	+	−	+
Root bark	+	+	+	+	+	+	+	−	+
Fruits	+	+	−	+	+	+	+	−	+
*Anonidium mannii *									
Leaves	+	−	**−**	−	+	+	+	+	+
*Gladiolus quartinianus *									
Whole plant	+	+	**−**	+	+	−	+	+	+
*Peperomia fernandopoiana *									
Whole plant	+	+	−	+	+	−	+	+	−
*Ricinodendron heudelotii *									
Leaves	+	−	−	−	+	+	+	−	+
Stem bark	+	+	−	+	+	−	+	−	−
*Vepris soyauxii *									
Leaves	+	+	−	+	+	+	+	+	+
Stem bark	+	+	+	+	+	+	+	−	+
Roots bark	+	+	−	+	+	+	+	−	+

(+): present; (−): absent.

**Table 3:** Cytotoxicity of the studied extracts towards sensitive and drug-resistant cancer cell lines and normal cells as determined by the resazurin assay.

Cell lines	Studied samples, IC_50 _values (*µ*g/mL)^a^ and degree of resistance (in bracket)			
	*Gladiolus quartinianus *(Whole plant)	*Vepris soyauxii *(Leaves)	*Anonidium mannii *(Leaves)	Doxorubicin
CCRF-CEM	10.57 ± 2.08	9.28 ± 1.01	17.32 ± 2.27	0.11 ± 0.01
CEM/ADR5000	26.14 ± 1.97 (2.47)	11.72 ± 1.43 (1.26)	16.44 ± 1.76 (0.95)	195.12 ± 14.30 (1772)
MDA-MB-231	16.11 ± 1.62	7.52 ± 0.84	12.65 ± 1.49	1.10 ± 0.01
MDA-MB-231-*BCRP *	29.6 ± 3.19 (1.49)	12.93 ± 1.69 (1.71)	32.02 ± 3.16 (2.53)	7.83 ± 0.01 (7.11)
HCT116 *p*53^+/+^	19.83 ± 1.66	8.59 ± 0.88	13.61 ± 1.79	1.43 ± 0.02
HCT116 *p*53^−/−^	22.15 ± 1.97 (1.12)	9.70 ± 0.72 (1.12)	∗ (>2.94)	4.06 ± 0.04 (2.84)
U87MG	∗	8.75 ± 1.21	22.25 ± 2.76	1.06 ± 0.03
U87MG*ΔEGFR *	34.01 ± 2.78 (<0.85)	4.09 ± 0.56 (0.47)	9.14 ± 1.77 (0.41)	6.11 ± 0.04 (5.76)
HepG2	∗ (n.a)	13.60 ± 1.22 (<0.34)	22.09 ± 2.42 (0.55)	1.41 ± 0.12 (<0.04)
AML12	∗	∗	∗	∗

^a^The degree of resistance was determined as the ratio of IC_50_ value of the resistant/IC_50_ sensitive cell line.

(∗): >40 *µ*g/mL; n.a: not applicable.
