# Supplementary material for: Cytotoxicity and Modes of Action of the Methanol Extracts of Six Cameroonian Medicinal Plants against Multidrug-Resistant Tumor Cells
Source: Evid Based Complement Alternat Med. 2013 Sep 23;2013:285903. doi: 10.1155/2013/285903 (PMC3794640; doi:10.1155/2013/285903)
Supplement: Supplementary file 1 — The three plants induced apoptosis and cell cycle arrest in leukemia CCRF-CEM cells. Upon 72 h treatment, Gladiolus quartinianus induced cell cycle arrest between G0/G1 and S phases, whilst Vepris soyauxii and Anonidium mannii induced arrest in G0/G1. [file 285903.f1.doc]

**Fig. S1.** Cell cycle distribution with leukemia CCRF-CEM treated with compounds **2-4** and doxorubicin at their IC_50_ values (See main document)

|  | **24 h** | **48h** | **72 h** |
| --- | --- | --- | --- |
| Control | 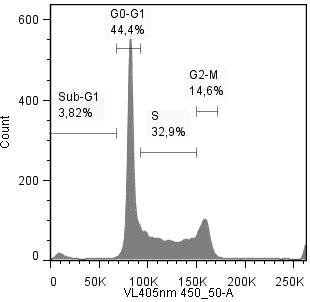 | 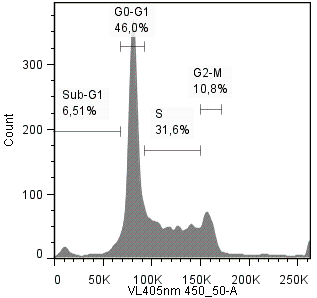 | 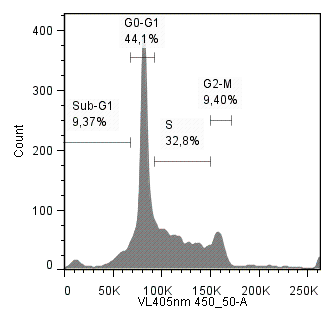 |
| Doxorubicin | 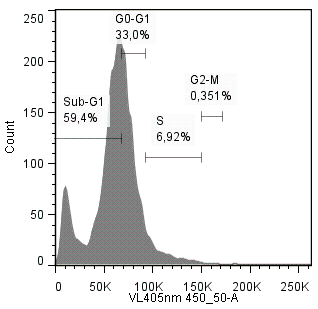 | 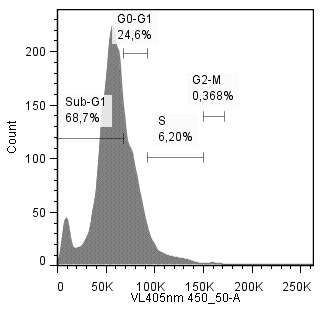 | 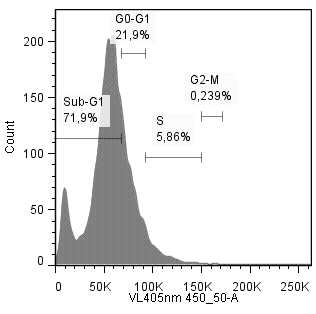 |
| *Gladiolus quartinianus* | 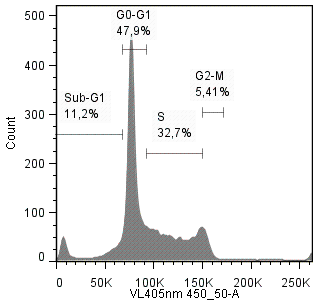 | 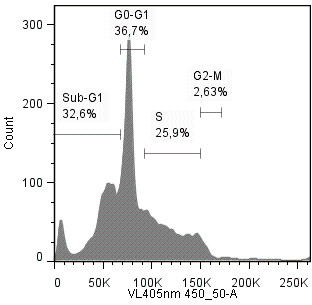 | 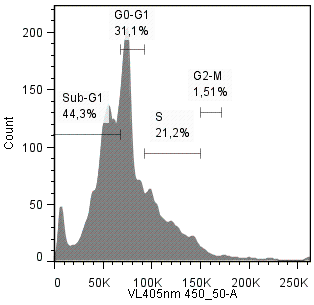 |
| *Vepris soyauxii* | 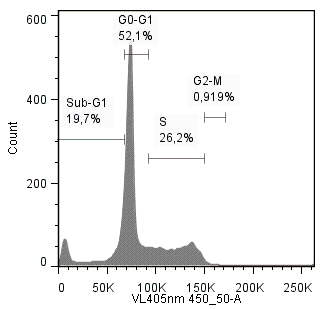 | 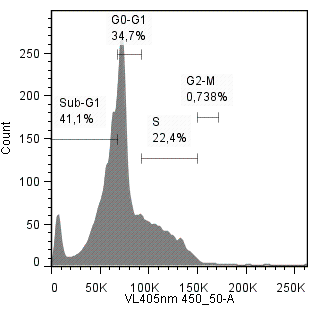 | 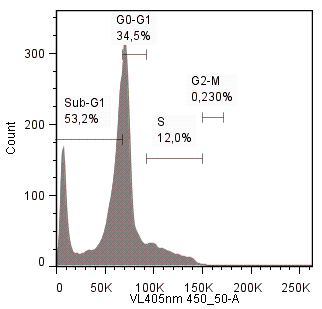 |
| *Anonidium mannii* | 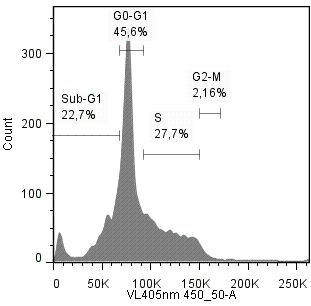 | 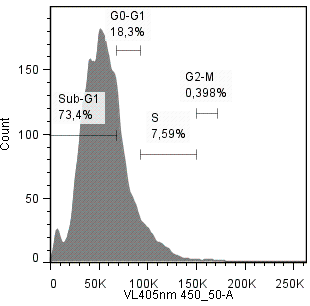 | 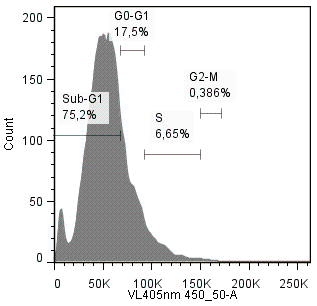 |
